# Operator’s eye lens dose in computed tomography–guided interventions

**DOI:** 10.1007/s00330-020-07576-0

**Published:** 2020-12-21

**Authors:** Siru Kaartinen, Minna Husso, Hanna Matikka

**Affiliations:** Department of Clinical Radiology, Kuopio University Hospital, Puijonlaaksontie 2, 70210 Kuopio, Finland

**Keywords:** Tomography, X-ray computed, Radiation protection, Lens, Crystalline

## Abstract

**Objectives:**

To survey (1) operator’s eye lens doses in typical computed tomography (CT)-guided interventions, (2) correlation between dose length product (DLP) and the operator’s dose, and (3) different ways for estimating the eye lens dose in clinical settings.

**Methods:**

Doses of 16 radiologists in 164 CT-guided interventional procedures were prospectively measured during a 6-month time period upon radioprotective garments and descriptive statistical outcomes were calculated. The correlations between DLP and measured doses were surveyed.

**Results:**

On average, the operator’s dose at the eye level (DEL, H_p_(0.07)) was 22 μSv per procedure and the personal equivalent dose H_p_(10) at the collar level was 21 μSv per procedure. The mean DLP of a procedure was 320 mGy cm, where 54% resulted from the fluoroscopy, the mean exposure time being 18 s. Based on the results, the operator’s DEL could be estimated from DLP using the equation DEL (μSv) = 0.10 μSv/mGy cm × patient fluoro DLP (mGycm) (*p* < 0.001), and the dose at the collar level (DCL) using the equation DCL (μSv) = 0.12 μSv/mGy cm × patient fluoro DLP (mGy cm) (*p* < 0.001). In addition, DEL (μSv) = 0.7 × DCL (μSv).

**Conclusions:**

The eye lens doses in CT-guided interventions are generally low even without protective equipment, and it is unlikely that the recommended annual equivalent dose limit of 20 mSv for the lens of the eye will be exceeded by conducting CT-guided interventions solely. Eye lens dose can be roughly estimated based on either DLP of the procedure or dose measured at the operator’s collar level.

**Key Points:**

*• Eye lens doses in CT-guided operations are generally low.*

*• It is unlikely that the ICRP recommendation of the yearly equivalent dose limit of 20 mSv will be exceeded by conducting CT-guided interventions solely.*

*• Magnitude of eye lens dose can be estimated based on either DLP of the procedure or dose measured at the operator’s collar level.*

## Introduction

Operators are exposed to variable levels of ionizing radiation at their work due to X-ray-guided patient interventions and examinations. It has been recognized that ionizing radiation may cause cataract: ocular lens opacity with visual impairment [1]. Previously, lens opacities were believed to occur after acute exposures to 0.5–2 Sv or more and vision-impairing cataracts after 5 Sv [1–3]. However, recent data from animal models and human populations suggest that the limit for the potentially harmful dose is considerably lower and could result in e.g. changes in contrast sensitivity, blurred vision, and loss of visual acuity [4–11]. Consequently, the International Commission on Radiological Protection (ICRP) published a recommendation in 2012 for a new dose threshold for visual-impairing cataracts of 0.5 Gy and lowered the recommendation for occupational dose limit for eye lens from 150 to 20 mSv per year [12]. Since then, the occupational exposure of the eye has gained attention related to e.g. cardiological procedures [13–17], hybrid operating rooms [18], and neuroradiological procedures [19]. However, until now, the eye doses in computed tomography (CT)-guided interventions have not been well documented regardless of routine use. In the literature review, only one paper [20] studying eye lens doses of the staff in CT-guided interventions was found. Thus, more knowledge about typical occupational eye doses from CT-guided interventions and about optimal ways to determine them are needed.

Typical CT-guided interventions are biopsies, drainages, and hyperthermal tumor therapies [21] in which a traditional CT scan is firstly used for planning the procedure. During the intervention, real-time fluoroscopic CT is used to provide the operator’s immediate feedback, i.e., for targeting a biopsy needle. In most procedures, the operator has to stay close to the patient while using fluoroscopy. In some procedures, it is possible to use protective extra shields like a lead glass shield, but in many cases only personal shielding like aprons and glasses can be employed. The durations of the procedures can vary extensively from minutes to hours where the fluoroscopic exposure time varies from seconds to minutes. Thus, in some cases, the operator’s radiation exposure can be notable.

In clinical radiology, different kinds of metrics and dosimeters are used to estimate and monitor radiation doses of personnel [21]. Equivalent dose H_T_ (mSv) is a measure of radiation dose to an organ or tissue (T) which also takes into account the quality of the radiation. The personal (P) dose equivalents H_p_(10) and H_p_(0.07) (mSv) are measures of radiation doses at 10 mm and 0.07 mm depth, respectively, from skin surface at the measurement site. H_p_(10) reflects the personal effective dose and is also called the deep dose equivalent and H_p_(0.07) is used as equivalent to the skin dose. To monitor the dose to the eye lens, measurement of H_p_(3) is recommended by ICRP. However, for now, very few physical dosimeters are available and/or calibrated to actually measure H_p_(3). For example in Finland, there is currently no certified dosimeter available for measuring the eye lens doses.

The purpose of this study was to survey (1) operator’s eye lens doses in typical CT-guided interventions, (2) correlation between DLP and operator’s dose, and (3) different ways for estimating the eye lens dose in clinical settings.

## Materials and methods

This prospective single-center study, in which the eye lens doses of operators were measured with multiple dosimeters during various CT-guided interventions, was approved by the institutional review board of Kuopio University Hospital. The study was conducted at the Department of Clinical Radiology in Kuopio University Hospital (Kuopio, Finland) where around 300 CT-guided operations are yearly conducted.

In this prospective study, dose measurements of operators were conducted in 164 CT-guided interventional procedures during a 6-month time period. The procedures included 59 epidural steroid injections, 57 lung biopsies, 16 bone biopsies, 16 drainages, eight pre-operative tumor marker placements, five soft tissue biopsies, and three tumor ablations. All 16 radiologists that performed CT-guided procedures were included in the study (12 male, 4 female). On average, the operators had 7 years of experience in CT-guided operations (range 1–20 years, median 6 years). The height of the operators was 177 cm in average (range 165–187 cm, median 178 cm). The operators wore thyroid shields and lead aprons in all procedures, and lead glasses in the majority of the procedures (70%). Only when the radiologist was able to stay behind the lead glass shield were radiation protective glasses not worn. The dosimeters were placed so that the personal protective garments did not cover them. The lead equivalence of the thyroid shield, lead apron, and lead glasses was 0.5 mm at 100 kV.

**Fig. 1:**
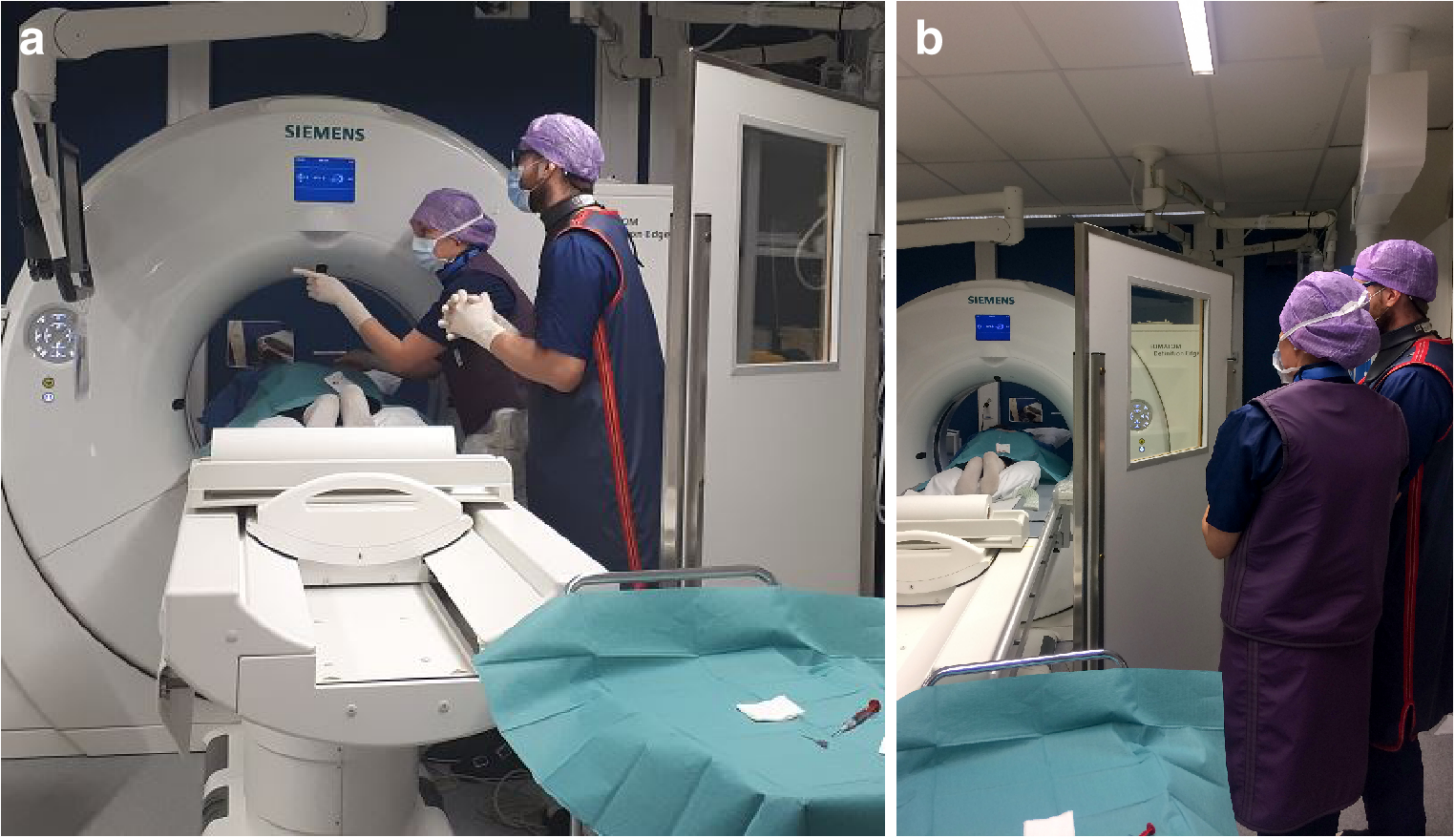
A typical positioning of the operators during a CT guided operation where (**a**) the operator needed to hold the instruments and stay approximately 30–50 cm from the fluoroscopy area and (**b**) the operators were able to stand behind the lead wall during fluoroscopy without having to constantly hold the instruments. The operator is on the left and the assisting operator on the right. In this particular procedure, the operator is not wearing lead glasses, the assistant operator is

The procedures were performed either on Somatom Edge (Siemens AG, 64 detector rows, 78-cm gantry opening) or Sensation 16 (Siemens AG, 16 detector rows, 70-cm gantry opening) multi-detector CTs. First, a planning CT scan was performed during which the personnel was not in the CT room. Based on the planning CT scan, an optimal route and accessories for the procedure were determined. Then, the operator, staying in the CT room, used CT fluoroscopy to guide the procedure (100–120 kV, 30–80 mAs, collimation 6 × 1.2 mm). The height of the patient table was 152 cm on average (range 126–172, median 156 cm). The operator’s distance from the patient and fluoro scan area varied depending on the type of the operation: In the majority of the procedures, the operator needed to hold the instruments and stay approximately 30–50 cm from the fluoroscopy area (Fig. 1a). In some of the procedures (most epidural steroid injections and some drainages and tumor biopsies), the operators were able to stand behind the lead wall (180 × 90 cm, 0.5 mm Pb at 100 kV) during fluoroscopy without having to constantly hold the instruments (Fig. 1b). After each procedure, the operator gave a subjective evaluation of the complexity of the procedure on a scale of 1–3 (1 easy, 2 moderate, 3 difficult).

The eye lens doses were measured using a prototype of an eye dosimetry headband using a Harshaw Extrad^TM^ dosimeter element which was calibrated for H_p_(3) measurements (Rotunda Scientific Technologies). Furthermore, as there was no certified method for eye lens dose measurements H_p_(3), routine dosimeters (measuring H_p_(0.07) and H_p_(10)) were also used in order to study the relationship between the doses measured with routinely used dosimeters and the eye lens dosimeter.

**Fig. 2:**
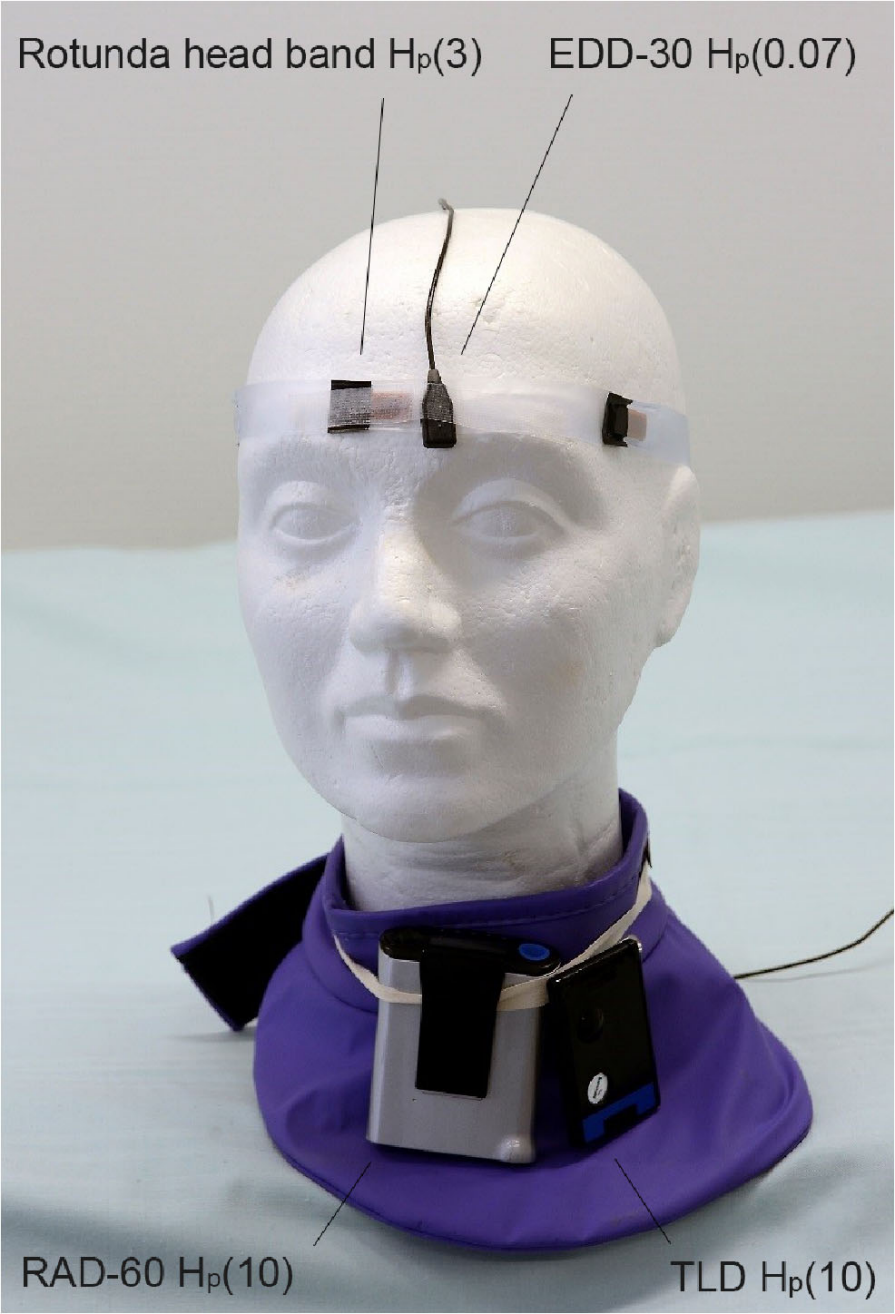
Placement of the dosimeters. Dose was measured with (1) a TLD attached to a headband at the middle of the forehead (Rotunda headband) (H_p_(3)) and with (2) EDD-30 placed on the forehead of the radiologist (H_p_(0.07)). Total dose was measured with (3) a regular TLD attached to the thyroid shield (H_p_(10)) and with (4) RAD-60 personal electronic dosimeter at collar level (H_p_(10))

During each procedure, the operator wore two dosimeters at eye level and two dosimeters at collar level (Fig. 2). The radiation doses were measured so that the protective garments did not cover the dosimeters. At the collar level, a regular passive personal thermo-luminescent detector (TLD, Doseco) was attached to the thyroid shield and used for measuring the cumulative personal doses (H_p_(10) and H_p_(0.07)). In order to measure the personal equivalent dose per procedure at the collar level, also an active RAD-60 personal electronic dosimeter (Mirion Technologies) was used. The eye lens dose per procedure was measured with an Educational Direct Dosimeter 30 (EDD-30, Unfors Instruments) and the sensor was placed on the forehead above the eyes. The EDD-30 was calibrated in terms of H_p_(0.07). Furthermore, a prototype headband (Rotunda Scientific Technologies) with a Harshaw Extrad^TM^ dosimeter element was used for measuring the cumulative eye lens dose for eight radiologists. The product incorporated a 300 mg/cm^2^ polytetrafluoroetylene filter and was calibrated for measuring H_p_(3). The headband was worn so that a middle TLD laid between the eyes on the forehead. The headband (with limited availability) was used only for the radiologists who conduct the majority of the procedures at the department. The number of different procedures performed by radiologists wearing Rotunda headband is given in Table 3. The calibration accuracies, energy responses, and angular dependencies of the dosimeters are given in Table 1.

**Table 1:** The placement, calibration error, energy response and angular dependency of the dosimeters

Dosimeter	Placement on the operator	Calibration error	Energy response	Angular dependency
TLD	Above the collar shield	H_p_(0.07) 5% and H_p_(10) 3% at 662 keV (Cs-137)	± 24% (20–1300 kV) (TLD data sheet)	< 60%
RAD-60	Above the collar shield	Less than 5% at 662 keV (Cs-137)	± 25% (60–1500 kV) (RAD-60 data sheet)	< 50% (± 75°)
EDD-30	Middle of the forehead	6% at 80 kV	± 25% (20–65 kV) (EDD-30 data sheet)	< 10% (± 75°)
Rotunda head band	Middle of the forehead (for 8 operators)	5% at 662 keV (Cs-137)	± 20% (20–662 kV) [22]	< 20% (± 60°)

The passive dosimeters were read in a company (Doseco Oy) that is a certified monitoring service company approved by the Finnish Radiation and Nuclear Safety Authority (STUK). The readout of the passive dosimeters (Rotunda headband with a Harshaw Extrad^TM^ dosemeter element and TLD dosimeters held above the thyroid shield) was done every month or every three months depending on the operator using the dosimeter: some of the operators are class A radiation workers whose dosimeters are read monthly and some operators are class B radiation workers whose dosimeters are read every three months. The background subtraction for the passive dosimeters was conducted at the same time. The readout of the active dosimeters (RAD-60 and EDD-30) was done after every procedure and no background subtraction was conducted.

The statistical analyses were done using SPSS 23.0.0.2 software (IBM). A power analysis was used for determining an adequate sample size. Quantitative parameters are expressed as mean values and ranges. Student’s *t* test was used for comparing the statistical differences between the groups. The Spearman correlation coefficient, with its corresponding *p* value, was used to examine the relationship between operator’s lens doses and the doses measured with various dosimeters, the fluoroscopy time, and the patient fluoroscopy DLP. Scatter plots with a regression line were used to illustrate correlations between quantitative parameters.

## Results

### Operator’s doses in various procedures

In procedures where the operator stayed behind the lead screen (*n* = 47), the mean dose per procedure was below the detection level of the dosimeters. These measurements were not included in the further description and analysis of results. The DLP was smallest in epidural steroid injections (183 mGy cm, range 19–527 mGy cm, median 154 mGy cm).

On average, the dose of the operator (working without a lead wall) at the eye level (H_p_(0.07)) was 22 μSv per procedure (range 0.04–89 μSv, median 15 μSv) (Table 2). The average personal equivalent dose H_p_(10) at the collar level was 21 μSv per procedure (range 1–128 μSv, median 12 μSv). The mean DLP of a patient was 320 mGy cm (range 57–1210 mGy cm, median 250 mGy cm), where 54% (174 mGy cm, range 14–962 mGy cm, median 115 mGy cm) resulted from the fluoroscopy with an average exposure time 18 s (range 13–107 s, median 13 s). The largest doses at the eye level resulted from tumor ablations, where the average dose (H_p_(0.07)) was 75 μSv (range 49–89 μSv). The detailed results are presented in Tables 2 and 3. Eighty-five procedures were performed from the right side of the CT gantry and 79 procedures from the left side. There was no difference in the measured doses H_p_(0.07 and 10) of the radiologist or the patient depending on the side (*p* > 0.1). The average estimated complexity of the procedures was 1.5 and there was no difference in the estimated complexity between the two devices used. The results are presented for both the devices together.

**Table 2:** The number of different procedures, mean (range) dose (μSv) of the operator measured with EDD-30 dosimeter at eye level, mean (range) dose (μSv) of the operator at collar level measured with RAD-60 dosimeter, mean (range) total dose length product (DLP, mGy cm), mean (range) DLP (fluoro only, mGy cm), mean (range) exposure time (s) (fluoro only), mean complexity of the procedure (1–3)

Procedure	Number of procedures	Mean dose of the radiologist (μSv) at eye level (measured with EDD-30)	Mean dose of the radiologist (μSv) at collar level (measured with RAD-60)	Total DLP of the patient (mGy cm)*	DLP of the patient, fluoroscopy mode only (mGy cm)	Exposure time (s)	Mean complexity of the procedure
Tumor ablation	3	75 (49–89)	128 (44–171)	908 (755–1210)	430 (215–586)	43 (33–58)	2.3
Other biopsies	5	30 (0–77)	35 (0–83)	544 (285–959)	414 (211–847)	28 (14–57)	1.8
Drainages	16	30 (0–55)	38 (0–123)	469 (132–1082)	283 (39–887)	18 (4–59)	2
Pre-operative tumor marking	8	25 (9–50)	25 (5–68)	350 (168–606)	206 (94–416)	20 (10–46)	2
Lung biopsies	57	20 (4–70)	23 (1–109)	262 (97–1141)	153 (44–962)	19 (5–107)	1.6
Epidural steroid injections	59	2 (0–25)	2 (0–19)	183 (19–527)	39 (3–166)	9 (2–30)	1.2
-Without lead shield	29	4 (0.1–22)	4 (0–14)	193 (57–527)	36 (5–157)	9 (3–30)	1.2
-With lead shield	30	**	**	174 (19–464)	41 (3–166)	10 (2–27)	1.2
Bone biopsies (with lead shield)	16	0.01 (0–0.04)	0.1 (0–1)	310 (99–896)	149 (50–516)	14 (3–51)	1.7
Total Without lead shield	164 116	14 (0–89) 22 (0.04–89)	17 (0–171) 21 (1–128)	284 (19–1210) 320 (57–1210)	140 (3–962) 174 (14–962)	16 (2–107) 18 (13–107)	1.8

*Total patient DLP contains the planning CT scan and fluoroscopy that was used during the operation

**The dose was below the detection level of the dosimeters

**Table 3:** The number of different procedures performed by radiologists wearing a Rotunda headband

Radiologist	Tumor ablation	Other biopsy	Drainage	Pre-operative tumor marking	Lung biopsy	Epidural steroid injection	Bone biopsy	Total amount of procedures
1	1	2	8	1	9			21
2	1		6	4	15			26
3		1		2	15			18
4		2				8	7	17
5						4		4
6						4		4
7						18		18
8						9		9

### Measuring and estimating the operator’s dose in CT-guided procedures

#### Measurements with active dosimeters

**Fig. 3:**
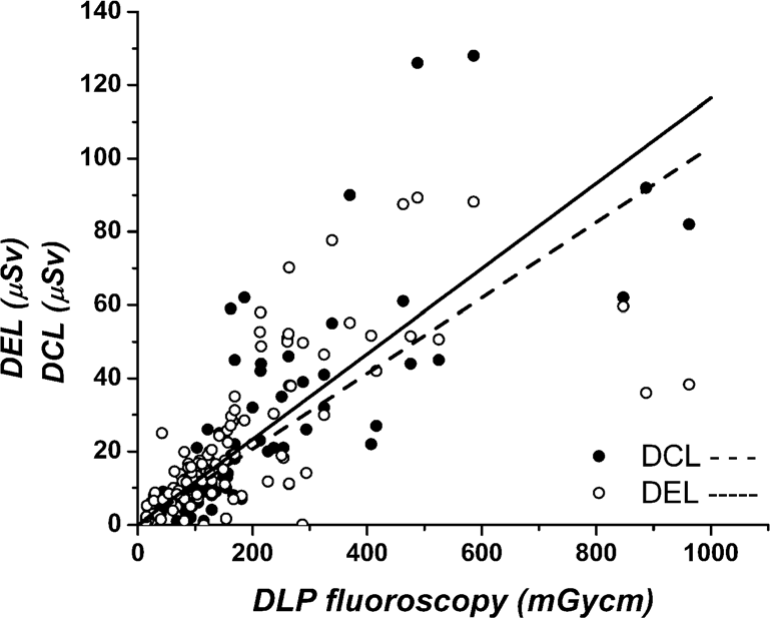
Scatter plots with regression line indicating correlation of DEL and DCL measured with EDD-30 (μSv) (white circle, solid line) and RAD-60 (μSv) (black circle, dashed line), respectively, with DLP from fluoroscopy (mGy cm)

There was a statistically significant correlation between the fluoroscopy DLP (mGy cm) and the dose of the operator measured at the eye level with the EDD-30 (H_p_(0.07), μSv) (correlation coefficient *r* = 0.78, *r*^2^ = 0.60, *p* < 0.001, *n* = 106, confidence interval S (95%) = 29, standard error of estimate 12 μSv, DEL/DLP range 0.0003–0.6; Fig. 3). Based on our results, the dose (H_p_(0.07)) at eye level (DEL) can be estimated from the DLP using the following Eq. 1:

1 $$ \mathrm{DEL}\ \left(\upmu \mathrm{Sv}\right)=0.10\ \upmu \mathrm{Sv}/\mathrm{mGy}\ \mathrm{cm}\times \mathrm{fluoro}\ \mathrm{DLP}\ \left(\mathrm{mGy}\ \mathrm{cm}\right) $$

The personal dose of the radiologist measured at collar level (DCL) with RAD-60 H_p_(10) also correlated significantly with the DLP from fluoroscopy mode (*r* = 0.81, *r*^2^ = 0.66, *p* < 0.001, S (95%) = 24.6, *n* = 106, standard error of estimate 14 μSv, DCL/DLP range 0.01–0.7; Fig. 3). Thus, the personnel dose (H_p_(10)) at collar level can be estimated using the following Eq. 2:

2 $$ \mathrm{DCL}\ \left(\upmu \mathrm{Sv}\right)=0.12\ \upmu \mathrm{Sv}/\mathrm{mGy}\ \mathrm{cm}\times \mathrm{fluoro}\ \mathrm{DLP}\ \left(\mathrm{mGy}\ \mathrm{cm}\right) $$

**Fig. 4:**
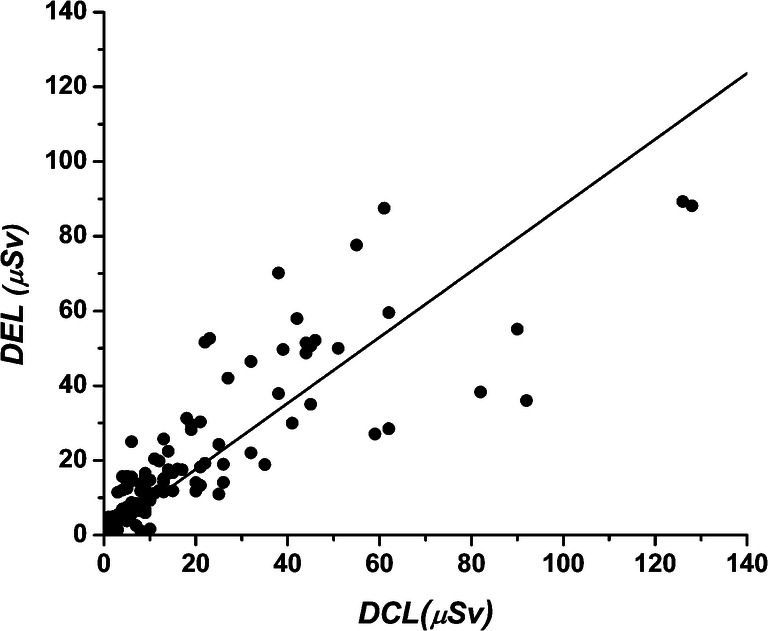
Dose measured with EDD (μSv) at eye level (DEL) and with RAD-60 (μSv) at collar level (DCL)

There was a statistically significant correlation between the doses measured with EDD (H_p_(0.07) μSv) at eye level and RAD-60 (H_p_(10), μSv) at collar level (*r* = 0.87, *r*^2^ = 0.76, *p* < 0.001, S (95%) = 22.8, *n* = 106, standard error of estimate 10 μSv, DEL/DCL range 0.04–4.8; Fig. 4) and the dose at eye level (DEL, H_p_(0.07)) can be estimated using Eq. 3:

3 $$ \mathrm{DEL}\ \left(\upmu \mathrm{Sv}\right)=0.7\times \mathrm{DCL}\ \left(\upmu \mathrm{Sv}\right) $$

#### Measurements with passive dosimeters

There was no statistically significant correlation between the measurements with Rotunda headband (middle dosimeter) and EDD-30 (Table 4, *p* = 0.889). There was no statistically significant correlation between the TLD at collar level and RAD-60 (Table 4, *p* = 0.123).

**Table 4:** Cumulative doses of the radiologists measured at the eye level with (a) Rotunda headband middle dosimeter and (b) EDD, and doses at collar level measured with (c) RAD-60 and (d) TLD (Hp(10)), (e) TLD (Hp(0.07)); *n* = 117

Radiologist	a. Headband H_p_(3) (μSv)	b. EDD H_p_(0.07) (μSv)	c. RAD-60 H_p_(10) (μSv)	d. TLD H_p_(10) (μSv)	e. TLD H_p_(0.07) (μSv)
1	810	580	690	870	640
2	870	650	670	900	420
3	380	420	330	630	540
4	5	0.2	1	0	0
5	0	0.7	1	0	0
6	55	30	20	0	0
7	0	0	0	0	0
8	20	40	30	0	10

## Discussion

The average dose at eye level H_p_(0.07) in a total of 164 CT-guided interventions was 22 μSv. Thus, it can be estimated that around 900 yearly procedures would be needed in order to exceed the ICRP recommendation of the annual eye lens dose limit of 20 mSv. This would require more than 17 CT-guided procedures in a week, per person. The largest doses at eye level resulted from tumor ablations (H_p_(0.07), 75 μSv on average) and the smallest in epidural steroid injections when the operator stayed behind a lead screen further from the patient (H_p_(0.07), result below the detection level of the dosimeters). Hence, it is generally safe to say that it is unlikely to exceed the annual eye lens dose recommendation by performing CT-guided operations only. All of the doses were measured upon protective garments, so the real eye lens dose when using e.g. lead glasses is even smaller.

On the other hand, the highest dose at eye level H_p_(0.07) was 89 μSv during a tumor ablation and around 4 similar procedures weekly would suffice to exceed the annual limit. This highlights the importance of (a) measuring personnel eye lens doses and (b) use of lead glasses also in CT-guided procedures, similar to other high-dose modalities.

Our results demonstrate that the operator’s dose at eye level and dose at collar level in CT-guided interventions can be roughly estimated from the patient dose-relevant parameter DLP using specific formulae. The placement of the dosimeter at collar level was based on a recommendation given by ICRP [23] and the placement of the dosimeter above the lead shield was based on instructions given by Finnish Radiation and Nuclear Safety Authority. It should be noted that the backscattered component is eliminated by the lead shield and is not included in the result. The placement of the dosimeter on the forehead of the operator was such that it was not directly covered by the lead glasses used by the operator. The formula to estimate the dose based on patient dose-relevant parameter DLP was almost the same for DEL and DCL. This highlights the fact that the accuracy of the estimation is better with actually measuring the dose at collar level compared to only relying on DLP data. The accuracy and robustness of this suggested method are a scope of more extensive studies. However, the formulae given in this paper may provide good general estimates for many practical purposes.

Our results showed a statistically significant correlation between the dose measured with EDD-30 at eye level and dose measured with RAD-60 at collar level. Our result is of similar magnitude as in a simulation study where eye dose was found to be 0.75× neck dose in interventional procedures [24]. This further highlights that although different dosimeters have different properties in terms of calibration, energy, and angular dependency, it is still possible to use commonly existing dosimeters for estimating the level of occupational eye lens dose.

The tumor ablations were rated more difficultly than average tumor ablations (2.3). The epidural steroid injections were rated to be easier than normal (1.2). These operations also gave the highest and lowest DLPs, respectively. In the future, the subjective scaling could be improved with quantitative complexity indices.

Eye lens doses in CT-guided interventions have been previously studied by Heusch et al [20]. In their study, the median total exposure of the eye lens was 3.3 μSv. In our study, the measured eye lens doses were higher which might be due to the differences in measurement site, fluoroscopic parameters, workflow, patient size, and difficulty of the procedures. Nevertheless, both studies demonstrate that performing only CT-guided operations, the yearly eye lens dose recommendation is unlikely exceeded.

There are a few limitations in our study. First, DEL was measured with Rotunda headband in 117 procedures altogether. However, only three out of eight radiologists performed procedures that resulted in substantial DELs which limited the feasibility of statistical methods on that data. There was, however, a relationship between the measurements with DEL and DCL which shows that the linkage should be studied in more detail with future measurements. Despite the difficulty comparing the groups, there can been seen a difference in the dose levels: the doses measured with TLD were higher compared to the doses measured with RAD-60. The operations were conducted with energies up to 120 kV, where the energy of the scattered photons is in the range of around 20–110 keV (mean 52 keV) [25]. There is a difference in the energy response of RAD-60 and TLD (see Table 1). As TLD is able to measure doses at lower energies, this could explain the higher doses measured with the TLD. In addition, the doses measured with the headband were higher compared to the EDD measurements. The energy dependence for EDD is less than 25% in the energy range of 20–65 kV. As the measurable energy range for the head band is wider (less than 20%, 20–662 keV), this might explain the results. In the future, more data points for passive dosimeters are needed to confirm the results. Secondly, numbers of some procedure types were small (*n* < 10). Both of these issues should be taken into account in future study designs.

The distance of the operator from the gantry and patient also affects the personnel doses notably as one of the factors affecting the radiation dose is the distance of the object from the source/scattering source. Thus, also the height of the operator and height of the patient table affect the result. As we concentrated on estimating the radiation dose in clinical settings, we have not evaluated this matter in our research in detail and this could be addressed in the future. In this practical study, the distances and operators’ movements were not controlled and were described only with limited accuracy. However, we believe that the practices in procedures are somewhat generic: the operator either stays next to the moving target or is able to stand further away behind a lead screen. Both of these cases have been taken into account in this study.

One potential limitation of this study is that it is based on data from a single center. This might diminish the generalizability of the results. In the future, it would be beneficial to gather data from other centers too.

In conclusion, eye lens doses in CT-guided operations are generally low and it is unlikely that the recommended annual equivalent dose limit of 20 mSv for the lens of the eye will be exceeded solely by conducting CT-guided interventions even without protective eyewear. Dose at eye level can be estimated using a formula based on either fluoro DLP or dose measured at collar level.

## Abbreviations

CT: Computed tomography
DCL: Dose at collar level
DEL: Dose at eye level
DLP: Dose length product
EDD: Educational Direct Dosimeter
H_p_(0.07): Dose equivalent at the depth of 0.07 mm
H_p_(10): Dose equivalent at the depth of 10 mm
H_p_(3): Dose equivalent at the depth of 3 mm
ICRP: International Commission on Radiological Protection
TLDs: Thermo-luminescent detector
